# The Role of Grass *MUTE* Orthologs in GMC Progression and GC Morphogenesis

**DOI:** 10.3389/fpls.2021.678417

**Published:** 2021-06-24

**Authors:** Laura Serna

**Affiliations:** Facultad de Ciencias Ambientales y Bioquímica, Universidad de Castilla-La Mancha, Toledo, Spain

**Keywords:** *FAMA*, *FOUR LIPS*, grasses, guard cells, guard mother cell, morphogenesis, *MUTE*, orthologs

## Abstract

Stomata arose about 400 million years ago when plants left their aquatic environment. The last step of stomatal development is shared by all plant groups, and it implies a symmetrical cell division from the guard mother cell (GMC) to produce two guard cells (GCs) flanking a pore. In Arabidopsis, the basic helix-loop-helix transcription factor MUTE controls this step, upregulating cell-cycle regulators of the GMC division, and immediately afterward, repressors of theses regulators like *FAMA* and *FOUR LIPS.* Recently, three grass *MUTE* orthologs (*BdMUTE* from *Brachypodium distachyon*, *OsMUTE* from rice, and *ZmMUTE* from maize) have been identified and characterized. Mutations in these genes disrupt GMC fate, with *bdmute* also blocking GC morphogenesis. However, because these genes also regulate subsidiary cell recruitment, which takes place before GMC division, their functions regulating GMC division and GC morphogenesis could be an indirect consequence of that inducing the recruitment of subsidiary cells. Comprehensive data evaluation indicates that *BdMUTE*, and probably grass *MUTE* orthologs, directly controls GMC fate. Although grass MUTE proteins, whose genes are expressed in the GMC, move between cells, they regulate GMC fate from the cells where they are transcribed. Grass *MUTE* genes also regulate GC morphogenesis. Specifically, OsMUTE controls GC shape by inducing *OsFAMA* expression. In addition, while SCs are not required for GMC fate progression, they are for GC maturation.

## Introduction

Plants conquered land over 470 million years ago (Edwards et al., 1998; Berry et al., 2010). This event was contemporaneous with a series of innovations, among them, the appearance of a water-repellent cuticle interrupted by tiny stomatal pores (Edwards et al., 1998; Berry et al., 2010). Stomatal pores, flanked by two kidney-shaped guard cells (GCs), allowed gas exchange between the plant and the atmosphere to perform photosynthesis with a minimal water loss. To date, no other structure has managed to replace them, although GC morphogenesis has evolved over time, with grasses developing dumbbell-shaped GCs, instead of kidney-shaped ones (Stebbins and Shah, 1960; Rudall et al., 2017; Hepworth et al., 2018; Nunes et al., 2019).

**FIGURE 1:**
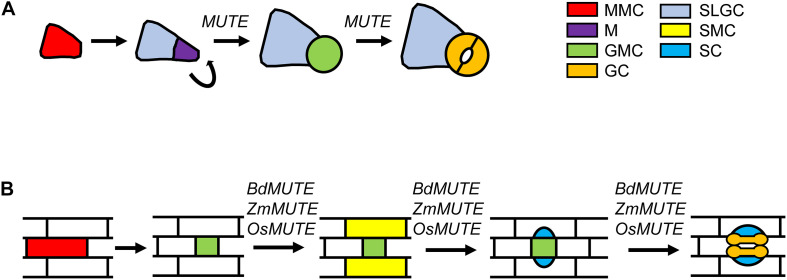
Steps regulated by *MUTE* and *MUTE* orthologs of grasses during stomatal development. **(A)** Stomatal development in Arabidopsis initiates when a protodermal cells acquires MMC identity. The MMC undergoes an asymmetric division that generates a small M and a larger SLGC. Ms usually reiterate their asymmetric divisions in an inward spiral. Ms activity stop when they assume GMC identity. GMCs divide symmetrically to produce the two kidney-shaped GCs. *MUTE* controls the transition from M to GMC, and the GMC division to produce a pair of kidney-shaped cells. **(B)** In grasses, stomatal development starts with an asymmetric division from an MMC that, in contrast with Arabidopsis, directly produces the GMC. Then, cells from files on either side of the GMC adopt SMC identity. SMCs divide asymmetrically to produce the two SCs making contact with the GMC. Once GMC is flanked by the SCs, it undergoes a symmetric division producing the two dumbbell-shaped GCs. Grass *MUTE* genes, in addition to control SMC identity and SCs formation, they also regulate GMC fate and GC morphogenesis. GC, guard cell; GMC, guard mother cell; M, meristemoid; MMC, meristemoid mother cell; SC subsidiary cell; SLGC, stomatal lineage ground cell; SMC, subsidiary mother cell.

In all plant species, stomatal development takes place through stereotyped patterns of cell divisions. The differences in these patterns among species give rise to a great diversity in the structure of the stomatal complexes. In the model plant Arabidopsis, protodermal cells commit to the stomatal lineage adopting, in a basipetal manner, the identity of meristemoid mother cell (MMC; Figure 1A; Peterson et al., 2010; Vatén and Bergmann, 2012). These MMCs undergo an asymmetric division to produce a smaller meristemoid (M) and a larger stomatal lineage ground cell (SLGC). Ms usually undergo additional self-renewing asymmetric divisions, in an inward spiral, until they become guard mother cells (GMCs). Then GMCs divide symmetrically to produce a pair of kidney-shaped GCs. SLGCs can differentiate into pavement cells, or they can assume an MMC fate producing secondary stomata. This cell division pattern differs from that taking place in grasses, where epidermal cells are organized in files, and stomatal development, which occurs only in some of them, proceeds along a spatiotemporal gradient with the earliest developmental stages occurring in the leaf base and proceeding as cells expand and differentiate toward the tip of the leaf (Stebbins and Shah, 1960). In this plant group, potential stomatal precursor cells proliferate in particular files and as these cells are pushed further up the leaf blade, some of them divide asymmetrically leading to a smaller GMC and a larger sister cell (Stebbins and Shah, 1960; Serna, 2011; Hepworth et al., 2018; Nunes et al., 2019; Figure 1B). Before GMC division, cells from files in either side of newly formed GMC acquire subsidiary mother cell (SMC) identity and divide asymmetrically. The smaller cells resulting from these cell divisions, which are always placed next to the GMC, differentiate as subsidiary cells (SCs). Following SCs recruitment, the GMC divides symmetrically, with the cell division plane being parallel to the main axis of leaf growth. This cell division, followed by a complex process of morphogenesis, yields two elongated, dumbbell-shaped GCs. The recruitment of SCs, together with the differentiation of dumbbell-shaped GCs, only takes place in this plant group.

**FIGURE 2:**
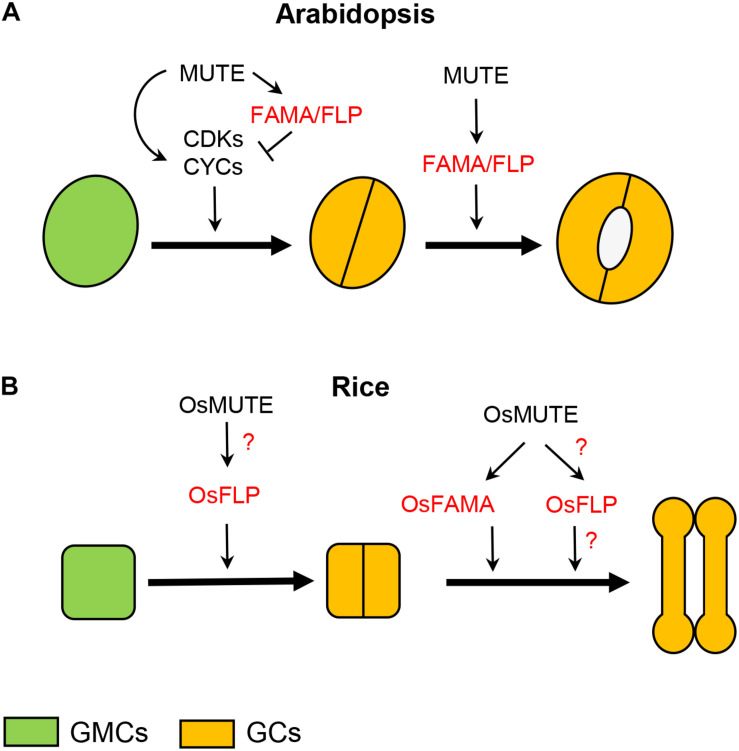
Role of *MUTE*, *FAMA*, *FLP*, and their orthologs in rice during GMC progression and GC morphogenesis. **(A)** In the GMC of Arabidopsis, MUTE positively regulates cell-cycle genes, but also, immediately after, repressors of them, among them *FAMA* and *FLP*. This makes possible that the GMC undergoes a single cell division. MUTE, by promoting *FAMA* and *FLP* expression, in addition to halt proliferative GMC divisions, controls GC differentiation. **(B)** In rice, OsMUTE guides GMC face by correctly orientating its cell division plane, perhaps by positively regulating *OsFLP*. OsMUTE also controls GC morphogenesis by promoting *OsFAMA* expression. OsMUTE may also regulate *OsFLP* to guide GC morphogenesis. It is not known what makes it possible for GMCs to undergo a single cell division. GC, guard cell; GMC, guard mother cell.

In Arabidopsis, the transition from GMC to paired GCs is regulated by *MUTE* (Han et al., 2018; Figures 1A, 2A and Table 1), which also controls the previous step, that is, the GMC formation from the last M (MacAlister et al., 2007; Pillitteri et al., 2007). The presence of arrested Ms, after an excess of self-renewing cell divisions, instead of stomata in *mute* loss-of-function mutants (MacAlister et al., 2007; Pillitteri et al., 2007), and the conversion of all epidermal cells to stomata in plants overexpressing *MUTE* (MacAlister et al., 2007; Pillitteri et al., 2007), are consistent with the functions attributed to this gene. *MUTE* encodes a basic-helix-loop-helix (bHLH) protein (MacAlister et al., 2007; Pillitteri et al., 2007), and its functions depend on its heterodimerization with the functionally redundantly bHLH proteins ICE1 (also known as SCREAM) and SCREAM2 (Kanaoka et al., 2008). Its expression, which overlaps with the localization of the protein encoded by this gene (Wang et al., 2019), is restricted to Ms and GMCs (MacAlister et al., 2007; Pillitteri et al., 2007). MUTE controls the last cell division of stomatal development directly upregulating cell-cycle regulators, and later transcriptional repressors of these cell-cycle regulators, like *FAMA* and *FOUR LIPS* (*FLP*) (Han et al., 2018 and references therein; Figure 2A). *FAMA*, which also encodes a bHLH protein that forms heterodimers with ICE1 and SCREAM2 (Ohashi-Ito and Bergmann, 2006; Kanaoka et al., 2008), not only ensures that GMCs undergo a single cell division, but also guides GC differentiation (Ohashi-Ito and Bergmann, 2006; Table 1). This gene is expressed and translated in GMCs and differentiating GCs (Ohashi-Ito and Bergmann, 2006). Independently of *FAMA*, the *MYB* gene *FLP*, which is strongly expressed in GMCs and in young GCs, together with its paralogous *MYB88*, also restricts GMC-division and guides GC differentiation (Lai et al., 2005; Table 1).

**TABLE 1:** Role of *MUTE, FAMA*, and *FLP*, and their orthologs in grasses.

Gene name	Species	Gene function	References
*MUTE*	*Arabidopsis thaliana* (Eudicot)	Transition from M to GMC, and from GMC to paired GCs	MacAlister et al., 2007; Pillitteri et al., 2007; Han et al., 2018
*FAMA*	*Arabidopsis thaliana* (Eudicot)	GMC and GCs identities	Ohashi-Ito and Bergmann, 2006; Han et al., 2018
*FLP*	*Arabidopsis thaliana* (Eudicot)	GMC and GCs identities	Lai et al., 2005; Han et al., 2018
*BdMUTE*	*Brachypodium distachyon* (Monocot, Poaceae)	Recruitment of SCs. GMC and GCs identities	Raissig et al., 2017
*ZmMUTE/BZU2*	*Zea mays* (Monocot, Poaceae)	Recruitment of SCs. GMC and GCs identities	Wang et al., 2019
*OsMUTE*	*Oryza sativa* (Monocot, Poaceae)	Recruitment of SCs. GMC and GCs identities	Wu et al., 2019
*OsFAMA*	*Oryza sativa* (Monocot, Poaceae)	GC morphogenesis	Liu et al., 2009; Wu et al., 2019
*OsFLP*	*Oryza sativa* (Monocot, Poaceae)	GMC and GCs identities	Wu et al., 2019

*GCs, guard cells; GMC, guard mother cell; M, meristemoid; SCs, subsidiary cells.*

Three grass *MUTE* orthologs have been recently isolated and characterized (Raissig et al., 2017; Wang et al., 2019; Wu et al., 2019; Table 1): *BdMUTE* from *Brachypodium distachyon*, *ZmMUTE* from maize and *OsMUTE* from rice. They also regulate stomatal development but in a very different way to *MUTE* (Raissig et al., 2017; Wang et al., 2019; Wu et al., 2019). OsMUTE and BdMUTE, like MUTE, associate with their orthologs of both SCREAM and SCREAM2 to control stomatal development, although there are differences in the function of these bHLH proteins between the grasses and Arabidopsis, and also within the grasses themselves (Kanaoka et al., 2008; Raissig et al., 2016; Wu et al., 2019). In contrast with *MUTE*, these grass *MUTE* orthologs induce the recruitment of SCs, and the proteins encoded by them move from the GMC, where they are expressed, to the SMCs (Raissig et al., 2017; Wang et al., 2019; Wu et al., 2019). This led to speculation that grass *MUTE* genes function in a non-cell-autonomous way, meaning that they influence adjacent SMC where they are not transcribed (Raissig et al., 2017; Wang et al., 2019; Wu et al., 2019; Serna, 2020). Mutations in grass *MUTE* orthologs, in addition to blocking SCs formation, also disrupt GMC fate (Raissig et al., 2017; Wang et al., 2019; Wu et al., 2019). In Brachypodium, it is known that mutations in *BdMUTE* not only block GMC fate but also GC morphogenesis (Raissig et al., 2017). However, given that the execution of the GMC fate takes place after the recruitment of the SCs, it is not known if the effect of *MUTE* orthologs during GMC division and GC differentiation is direct or, conversely, a consequence of their requirement in the previous step. Here, I delve into the possible function of grass *MUTE* genes in GMC fate progression and GC differentiation. The emerging picture unravels that they control GMC fate in an autonomous manner. They also regulate GC morphogenesis. In addition, in rice, GC morphogenesis takes place through positive regulation of *OsFAMA* by OsMUTE. Moreover, several observations strongly suggest that SCs formation is not required for grass *MUTE* genes to trigger the GMC division, but for GC maturation.

## *BdMUTE* Controls Autonomously GMC Fate

In Arabidopsis, *MUTE* promotes both the transition from the M to the GMC and the symmetric division of the GMC to produce two paired GCs (MacAlister et al., 2007; Pillitteri et al., 2007; Han et al., 2018). Ms appear to be absent in grasses, where a single asymmetric division from the MMC directly produces the immediate stomatal precursor (Nunes et al., 2019; Serna, 2020). This stomatal precursor, the GMC, divides symmetrically to produce the two GCs. Do grass *MUTE* genes regulate the transition from GMC to the paired GCs as *MUTE* does in Arabidopsis?

Most GMCs (70%) of *bdmute* divide symmetrically with their division plane orientating like those of wild-type plants, but to produce dicot-like stomata (Raissig et al., 2017). The remaining of GMCs (around 30%) of this mutant do not produce stomata (Raissig et al., 2017). They fail to specify the orientation of the GMC division plane and/or undergo excessive and randomly oriented cell divisions (Raissig et al., 2017). These results indicate that *BdMUTE*, in a redundant manner with other factors, controls GMC fate. Given that *bdmute* is completely devoid of SCs, its ability to develop stomata are telling us that *BdMUTE*, together with unknown factors, regulates autonomously, that is, in the cells in which it is made, GMC fate.

In contrast to *bdmute*, both *bzu2-1* and *c-osmute*, with loss-of-function mutations in *ZmMUTE* and *OsMUTE* respectively, completely lack stomata (Wang et al., 2019; Wu et al., 2019). Instead, these mutants produce GMCs that undergo excessive, randomly oriented and/or asymmetric divisions, which give rise to short columns of elongated cells (Wang et al., 2019; Wu et al., 2019; Buckley et al., 2020; Serna, 2020). While in *c-osmute* these columns consist of two cells, in *bzu2-1* can appear up to four cells. Interestingly, *bzu2-1*, which develops a small percentage (4.61%; *n* = 802) of complexes with one SCs, does not develop GCs (Wang et al., 2019). This observation suggests that, in maize, GMC fate progression does not depend on SCs formation. Then *BdMUTE*, and probably grass *MUTE* orthologs, controls GMC fate in a fully autonomous manner, and not by inducing a signaling from SCs.

But how do grass *MUTE* genes control GMC division? In Arabidopsis, cyclin-dependent kinase complexes consisting of a CYCA2s and CDKB1;1 positively regulate GMC division (Boudolf et al., 2009; Vanneste et al., 2011). CYCD5;1, which interacts with CDKA1;1 (Boruc et al., 2010), also promotes GMC division (Han et al., 2018). The same happens with CYCD7;1 together with CDKB1, which also executes GMC division (Weimer et al., 2018). Upstream of these complexes is MUTE, which directly upregulates the expression of the genes encoding for these cell cycle regulator proteins (Han et al., 2018; Weimer et al., 2018). Later, FLP, whose expression is positively regulated by MUTE (Han et al., 2018), represses *CDKB1;1* expression, and GMC division, by binding to a *cis*-regulatory region in its promoter (Xie et al., 2010). Like *CDKB1;1*, *CDKA;1* is also a direct target of FLP/MYB88, which bind to its promoter (Yang et al., 2014). FLP/MYB88 also repress *CYCD7;1* expression (Weimer et al., 2018). This makes possible that GMCs undergo a single cell division. *FAMA*, whose expression is also induced by MUTE (Han et al., 2018), may also negatively regulate *CDKB1;1* to halt cell division (Boudolf et al., 2004). FAMA also binds to the *CYCD7;1* promoter to restrict *CYCD7;1* expression (Weimer et al., 2018). In contrast to Arabidopsis, rice has only one ortholog to *CYCA2s*, named *OsCYCA2;1* (La et al., 2006; Qu et al., 2018). OsCYCA2;1 forms a complex with OsCDKB1, which is the ortholog of Arabidopsis CDKB1;1 (Qu et al., 2018). This complex, in contrast to those between CYCA2s and CDBK1;1, does not regulate GMC divisions, but it controls the previous step that generates the GMC (Qu et al., 2018). Although we know the targets of MUTE, and of its downstream components FLP and FAMA, to control GMC fate, the same does not happen for OsMUTE and OsFLP. The only thing we know now is that *OsMUTE* regulates GMC division in a different way than *MUTE* does in Arabidopsis.

## OsMUTE Induces GC Morphogenesis Positively Regulating *OsFAMA* Expression

*BdMUTE* not only controls GMC fate but also GC morphogenesis as shows the fact that *bdmute* develops dicot-like stomata. Does this regulation of GC shape extend to the other grass *MUTE* genes? Or, on the contrary, is it exclusive to Brachypodium and perhaps lost with the domestication of grasses?

In the GMC of Arabidopsis, MUTE not only positively regulates cell-cycle genes (Han et al., 2018; Weimer et al., 2018; Figure 2A), but also the transcriptional repressors of theses cell-cycle genes (Han et al., 2018; Figure 2A). Among these repressors is *FAMA* (Han et al., 2018). Loss-of-function *fama* mutants fail to develop stomata, and instead they produce clusters of small and narrow cells named *fama* tumors (Ohashi-Ito and Bergmann, 2006), and overexpression of this gene converts all epidermal cells to unpaired GC-like cells (Ohashi-Ito and Bergmann, 2006). Thus, *FAMA* in addition to halt proliferative GMC divisions, induces GC morphogenesis (Ohashi-Ito and Bergmann, 2006; Figure 2A). This network started by MUTE ensures that GMCs undergo a single division producing the paired kidney-shaped GCs (Han et al., 2018). Analysis of relative expression of *OsFAMA* in *c-osmute* showed that it is significatively smaller than that in wild-type plants, indicating that, like in Arabidopsis (Han et al., 2018), OsMUTE induces *OsFAMA* expression (Wu et al., 2019), more probably in GMC and young GCs. Agree with this, RNA *in situ* hybridization determined the localization of *OsFAMA* transcript in the leaf epidermis of the sheath elongation zone (Liu et al., 2009), where GMC division and GC differentiation take place. However, the function of *FAMA* and *OsFAMA* does not seem identical: while loss-of-function mutations in *FAMA* induce *fama* tumors (Ohashi-Ito and Bergmann, 2006), those in *OsFAMA* usually result in the formation of stomata with box-shaped GCs instead of dumbbell-shaped ones (Liu et al., 2009; Wu et al., 2019). GMCs of *c-osfama* do not undergo extra cell divisions. So that while *FAMA* controls both GMC division and GC morphogenesis, *OsFAMA* only regulates GC differentiation (Figure 2). Agree with this, the expression of *ProFAMA:OsFAMA* in the Arabidopsis *fama-1* mutant induces GC differentiation but does not prevent stomatal cluster formation (Liu et al., 2009). In contrast, the expression under the control of *FAMA* promoter of the *Solanum lycopersicum* ortholog of *FAMA* (*SolycFAMA*) in *fama-1* complements the two defects of *fama-1*, preventing stomatal clusters formation and triggering GC differentiation (Ortega et al., 2019). This suggests that *OsFAMA*, and perhaps *FAMA* orthologs from grasses, has lost its ability to regulate GMC fate. The divergence between *FAMA* and *OsFAMA* is also evident when comparing their overexpression phenotypes: while ectopic *FAMA* expression is sufficient to confer GC character (Ohashi-Ito and Bergmann, 2006), ectopic expression of *OsFAMA* is not (Wu et al., 2019). Occasionally, *osfama* develops stomata devoid of one SC, suggesting that *OsFAMA* contributes to the recruitments of SCs (Wu et al., 2019). The presence of SCs in *osfama* is telling us that GC morphogenesis, at least in rice, does not depend on a mechanical force generated by the SCs. Although the functions of *FAMA* and *OsFAMA* are not identical, both MUTE and OsMUTE control GC morphogenesis by regulating *FAMA* and *OsFAMA*, respectively (Figure 2). The role of *MUTE* orthologs in GC morphogenesis is not, therefore, exclusive to Brachypodium, but extends, at least to rice, and probably to the remaining grasses.

MUTE also represses GMC division upregulating the expression of the transcriptional repressor of regulatory genes of the cell cycle *FLP* (Han et al., 2018), with loss-of-function mutations in both *FLP* and its paralogous *MYB88* resulting in exaggerated stomatal cluster with undifferentiated stomatal precursor cells (Lai et al., 2005). Previous studies have shown that *FLP* and *MYB88* function independently of *FAMA* (Ohashi-Ito and Bergmann, 2006). Mutations in *OsFLP* disrupt the orientation of the GMC division plane and GC differentiation (Wu et al., 2019), but in contrast to those in *FLP* and *MYB88*, they do not induce extra GMC divisions. Then, OsMUTE may regulate the orientation of the GMC division plane by regulating *OsFLP* expression (Figure 2B). Thus, it is not clear how grasses ensure that GMCs undergo a single cell division. We also do not know if the differences between *FAMA*/*FLP* and *OsFAMA/OsFLP* extend to the rest of grass *FAMA/FLP* orthologs.

## SCs Are Required for GC Morphogenesis

*OsMUTE* promotes GC morphogenesis producing dumbbell-shaped GCs in rice leaves (Wu et al., 2019). Surprisingly, the stomata placed on rice coleoptiles are like those of Arabidopsis and quite different from those in rice leaves (Guo et al., 2016). What prevents the coleoptile GCs from undergoing the morphogenesis process that gives rise to dumbbell-shaped GCs? The stomatal complexes of rice coleoptiles not only consist of kidney-shaped GC pairs, but they are anomocytic, and therefore devoid of SCs (Guo et al., 2016). Then, one possibility is that SCs, which do not seem to be required for GMC division, are for GC morphogenesis.

In rice leaves, OsMUTE moves from GMC, where its gene is transcribed (Liu et al., 2009; Wang et al., 2019), to epidermal cells of neighboring files, where it is likely to regulate the transcription of genes required for SCs recruitment (Wang et al., 2019; Serna, 2020). MUTE, whose gene is expressed in GMCs (MacAlister et al., 2007; Pillitteri et al., 2007), does not move from GMC to surrounding epidermal cells (Wang et al., 2019). In agreement with this, Arabidopsis does not recruit SCs, or its GCs undergo the morphogenesis process typical of the GCs of grasses. *OsMUTE* is also expressed in coleoptiles of rice (Guo et al., 2016). An attractive hypothesis lies in the inability of movement of OsMUTE from GMC to its adjacent epidermal cells placed on neighboring files, preventing SCs formation, and consequently GC morphogenesis. Alternatively, OsMUTE may move among cells but its function that induces the recruitment of lateral SCs may be blocked in coleoptiles.

The development of dicot-like stomata in coleoptiles of rice suggests that signals emanating from SCs trigger GC morphogenesis in rice leaves. But what is the molecular nature of these signals? The role of *OsFLP* in GC morphogenesis is unclear, but *OsFAMA*, positively regulated by OsMUTE, promotes GC morphogenesis (Wu et al., 2019). OsMUTE may regulate *OsFAMA* from SCs and, consequently, in a non-autonomous way, by inducing the expression of unknown genes. What seems to be clear is that SCs are required for GC morphogenesis. Agree with this view, *MUTEp:OsMUTE* expression partially complements the defects of *mute-1* by inducing the formation of kidney-shaped GCs from some stomatal precursor (Liu et al., 2009), but is not capable of inducing the differentiation of dumbbell-shaped GCs in the absence of SCs. Like *OsMUTE*, *ZmMUTE* driven by the *MUTE* promoter in *mute-1* produces kidney-shaped GCs from some stomatal precursors (Liu et al., 2009), but it is not capable of producing grass stomata or SCs.

*MUTE* and grass *MUTE* retain the control of GMC division, but they have also diverged, with grass *MUTE* acquiring two new functions: the recruitment of SCs and the production of dumbbell-shaped GCs. It is time to speculate that the grass stomata have evolved from those of plants with kidney-shaped GCs, and through a mechanism that involves the intercellular movement of grass MUTE. At an intermediate point of this evolutionary path is *Flagellaria indica*, which is closely related to grasses, and exhibits intermediate morphologies in its GCs, neither dumbbell nor kidney-shaped ones (Sack, 1994). Because *Flagellaria indica* exhibits SCs like those of grasses, that is, its complexes are paracytic-non-oblique (Sack, 1994; Rudall et al., 2017), it is likely that SCs only trigger the first steps of GC morphogenesis of grasses.

## Concluding Remarks

*BdMUTE*, in addition to recruit SCs, controls GMC fate in a fully autonomous manner. Although possibly grass *MUTE* orthologs also autonomously control GMC fate, experimental data are necessary to confirm it. Interestingly, the *bdmute* incomplete penetrance unravels that unknown factors trigger stomatal formation in this mutant (Nunes et al., 2019; Serna, 2020). The full disruption of GMC fate in both *osmute* and *bzu2-1* suggests that these unknown genes regulating GMC fate in Brachypodium may have been blocked with the agricultural practices (Ohashi-Ito and Bergmann, 2006; Serna, 2020). The isolation and characterization of additional grass *MUTE* genes from both domesticated and wild plants will be essential to determine whether there is a direct link between *BdMUTE* divergence and the human influence on agriculture.

Grass *MUTE* genes also control GC morphogenesis. In rice, the proteins encoded by these genes do it, like in Arabidopsis, by positively regulating *OsFAMA* expression. Because *OsFLP* controls the orientation of the GMC division plane (Wu et al., 2019), perhaps positively regulated by OsMUTE, its possible role during GC morphogenesis is unclear. Analysis of the morphogenesis of the GCs of *osflp* produced by correctly orientated GMC divisions, will help to deep into the function/s of *OsFLP* and to unravel how much it has diverged from *FLP.* It is important to note that while *FAMA* and *FLP*, regulated by MUTE, in addition to controlling GC differentiation, also ensure that GMCs undergo a single division (Lai et al., 2005; Ohashi-Ito and Bergmann, 2006; Han et al., 2018), *OsFAMA* and *OsFLP* do not ensure the repression of extra GMCs division. So far, we do not have any information about the function/s of *FAMA* and *FLP* genes in Brachypodium and maize. The analysis of the *FAMA* and *FLP* orthologs function/s in these plant species will let us know if the differences in *FAMA* and *FLP* functions between Arabidopsis and rice extend to the rest of grasses.

Finally, the presence of stomata like those of Arabidopsis in rice coleoptiles questions the role of *OsMUTE*/*OsFAMA* in this embryonic organ and suggests that SCs are required for GC morphogenesis. We could be close to revealing the origin of the peculiar and highly efficient stomata of grasses, which seems to be related to the intercellular movement of grass MUTE. This unique and highly efficient structure is likely to have contributed, 30–45 million years ago, to the successful expansion of this plant group (Kellogg, 2001; Hetherington and Woodward, 2003; Chen et al., 2017).

## Author Contributions

LS wrote the manuscript and designed the figures.

## Conflict of Interest

The author declares that the research was conducted in the absence of any commercial or financial relationships that could be construed as a potential conflict of interest.
